# 4-[(Benzyl­amino)­carbon­yl]-1-methyl­pyridinium halogenide salts: X-ray diffraction study and Hirshfeld surface analysis

**DOI:** 10.1107/S2056989021013505

**Published:** 2022-01-07

**Authors:** Svitlana V. Shishkina, Anna M. Shaposhnik, Vyacheslav M. Baumer, Vitalii V. Rudiuk, Igor A. Levandovskiy

**Affiliations:** a SSI "Institute for Single Crystals", NAS of Ukraine, 60 Nauky ave., Kharkiv, 61001, Ukraine; b V.N. Karazin Kharkiv National University, 4 Svobody sq., Kharkiv, 61022, Ukraine; c Farmak JSC, 63 Kyrylivska str., Kyiv, 04080, Ukraine; d Kyiv National Technical University of Ukraine, 37 Pobedy ave., Kyiv, 03056, Ukraine

**Keywords:** 4-[(benzyl­amino)­carbon­yl]-1-methyl­pyridinium, mol­ecular structure, crystal structure, Hirshfeld surface analysis

## Abstract

Two salts of 4-[(benzyl­amino)­carbon­yl]-1-methyl­pyridinium (**Am**) with chloride (C_14_H_15_N_2_O^+^·Cl^−^) and bromide (C_14_H_15_N_2_O^+^·Br^−^) anions were studied and compared with the iodide salt. **AmCl** crystallizes in the centrosymmetric space group *P*2_1_/*n* while **AmBr** and **AmI** form crystals in the Sohncke space group *P*2_1_2_1_2_1_. Crystals of **AmBr** are isostructural to those of **AmI**. The cation and anion are bound by an N–H⋯Hal hydrogen bond. Hirshfeld surface analysis was used to compare different types of inter­molecular inter­actions in the three structures under study.

## Abstract 2

The ability of 4-[(benzyl­amino)­carbon­yl]-1-methyl­pyridinium to form salts with halogenide anions was studied and Hirshfeld surface analysis to identify inter­molecular inter­actions was performed.

## Chemical context

Organic salts are of great importance for the pharmaceutical industry (Stahl & Wermuth, 2002 ▸). Many drugs are produced in the form of salts because of their higher solubility as compared to neutral compounds. The pharmacokinetic properties may be modified by the choice of counter-ion (Guerrieri *et al.*, 2010 ▸; He *et al.*, 2018 ▸). Therefore, the study of the ability of an active pharmaceutical ingredient to form salts with different ions is an actual problem.

4-[(Benzyl­amino)­carbon­yl]-1-methyl­pyridinium iodide is known as a multimodal anti­viral drug (Buhtiarova *et al.*, 2003 ▸; Frolov *et al.*, 2004 ▸; Boltz *et al.*, 2018 ▸; Cocking *et al.*, 2018 ▸). This salt crystallized in the *P*2_1_2_1_2_1_ ortho­rhom­bic space group and was studied by single-crystal X-ray diffraction, powder diffraction, IR spectroscopy and DSC (Drebushchak *et al.*, 2017 ▸). Screening varying different solvents and crystallization conditions did not reveal the formation of any other polymorphs. 
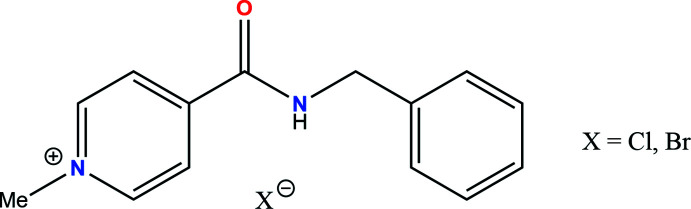


In the present work we studied salts of the 4-[(benzyl­amino)­carbon­yl]-1-methyl­pyridinium cation with chloride and bromide anions and compared their mol­ecular and crystal structures with that of the iodide salt.

## Structural commentary

Usually organic salts are obtained following hydrogen transfer within an acid–base pair. The equilibrium between the neutral acid–base pair and their cation–anion pair depends on external conditions such as temperature, concentration, nature of solvent, *etc* (Stahl & Nakano, 2002 ▸). As a result, organic cations formed upon protonation are not stable and can be deprotonated. The quaternization of the pyridine nitro­gen atom also results in cation formation (Wei *et al.*, 2018 ▸). However, such a cation is much more stable than its protonated analogue and can form salts with different anions.

**Figure 1:**
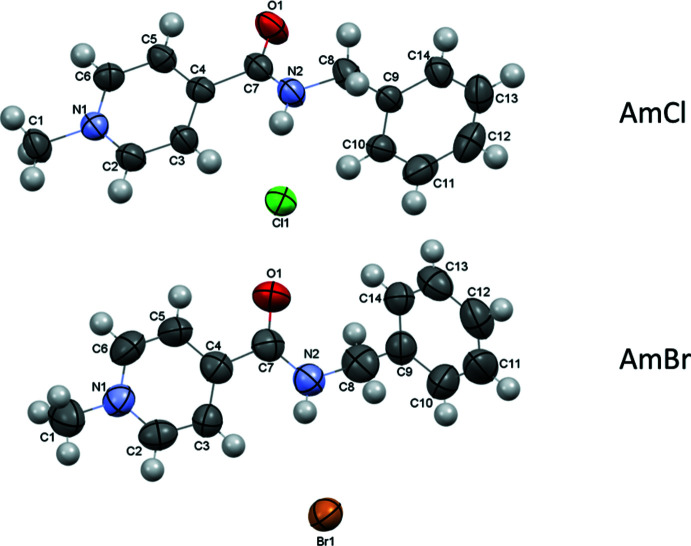
Mol­ecular structures of the title 4-[(benzyl­amino)­carbon­yl]-1-methyl­pyridinium halogenide salts. Displacement ellipsoids are shown with 50% probability level.

The organic cation is formed due to the quaternization of the pyridine moiety in the two salts under study (Fig. 1 ▸). The positive charge is located at the pyridine nitro­gen atom. The carbamide group and the pyridine ring are slightly non-coplanar in the chloride salt and coplanar in the bromide salt [the C5—C4—C7—O1 torsion angle is −13.3 (4)° in **AmCl** and −1.4 (16)° in **AmBr]**. The intra­molecular contacts H2⋯C3 = 2.57 Å, H2⋯H3 = 2.05 Å in **AmCl** and H2⋯C3 = 2.65 Å, H2⋯H3 = 2.16 Å in **AmBr** are shorter than the sums of the corresponding van der Waals radii (H⋯C = 2.87 Å and H⋯H = 2.34 Å; Zefirov, 1997 ▸) and point to a steric repulsion between the carbamide and pyridine fragments in the cations of **AmCl** and **AmBr**. The phenyl fragment of the benzyl substituent is positioned orthogonally to the carbamide unit and rotated around the N2—C8 bond [the C7—N2—C8—C9 torsion angle is −88.1 (4)° in **AmCl** and 93.5 (12)° in **AmBr** while the N2—C8—C9—C10 torsion angle is −24.3 (4)° in **AmCl** and 103.8 (12)° in **AmBr**].

The **AmCl** salt crystallizes in the centrosymmetric *P*2_1_/*n* space group while the **AmBr** salt crystallizes in the Sohncke space group *P*2_1_2_1_2_1_, similar to the **AmI** salt (Drebushchak *et al.*, 2017 ▸). The cation does not contain an asymmetric atom.

## Supra­molecular features

**Figure 2:**
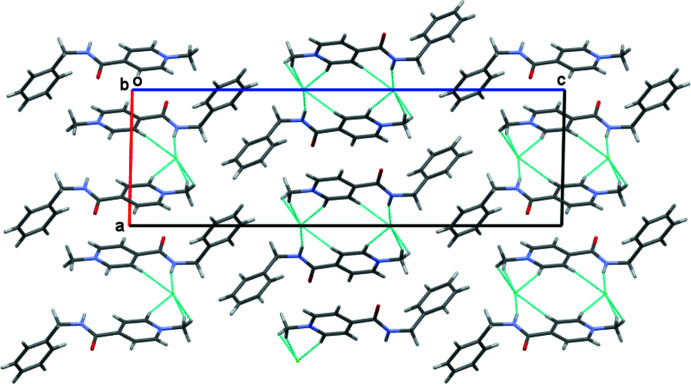
Crystal structure of 4-[(benzyl­amino)­carbon­yl]-1-methyl­pyridinium chloride. *X*—H⋯Cl hydrogen bonds are shown as dashed cyan lines.

**Figure 3:**
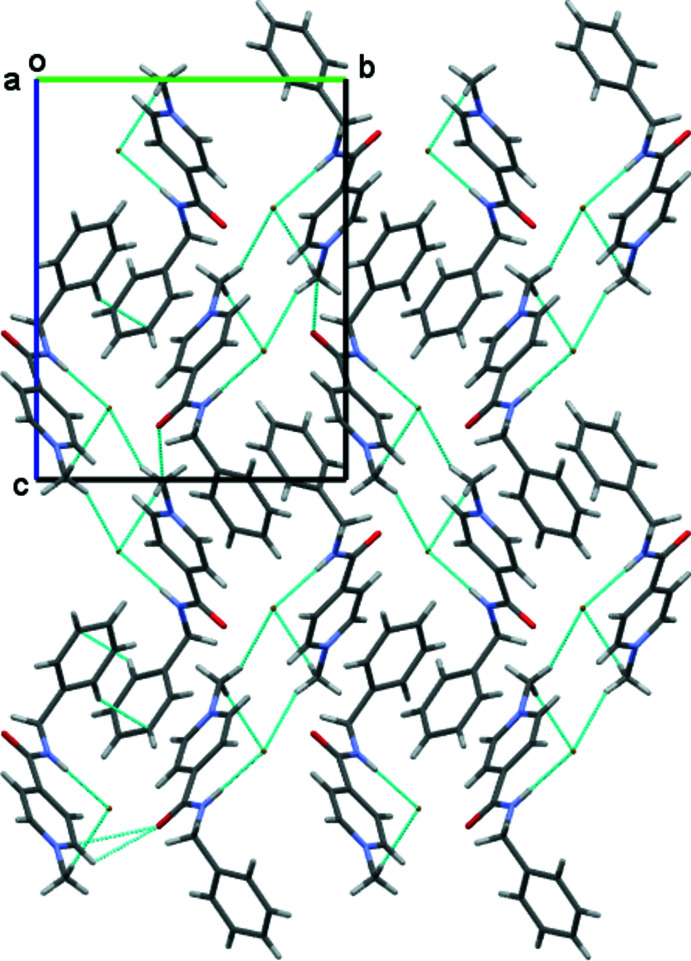
Crystal structure of 4-[(benzyl­amino)­carbon­yl]-1-methyl­pyridinium bromide. *X*—H⋯Br hydrogen bonds are shown as dashed cyan lines.

Analysis of the inter­molecular inter­actions revealed that an N—H⋯Hal inter­molecular hydrogen bond is present in both of the salts under study (Tables 1 ▸ and 2 ▸). This hydrogen bond is strongest in the **AmCl** salt as a result of the higher negativity of chloride anions as compared to bromide and iodide counter-ions. In addition, a set of C—H⋯Cl’ inter­molecular hydrogen bonds is found in **AmCl** (Fig. 2 ▸) while only two C—H⋯Hal’ hydrogen bonds are present in the crystal structure of **AmBr** (Figs. 3 ▸ and 4 ▸; Tables 1 ▸ and 2 ▸). Generally, the presence of pyridine and benzene rings in a mol­ecule can lead to the formation of π–π stacking inter­actions in the crystalline phase. However, no such stacking inter­actions were found in the **AmCl** and **AmBr** crystals.

## Hirshfeld surface analysis

**Figure 4:**
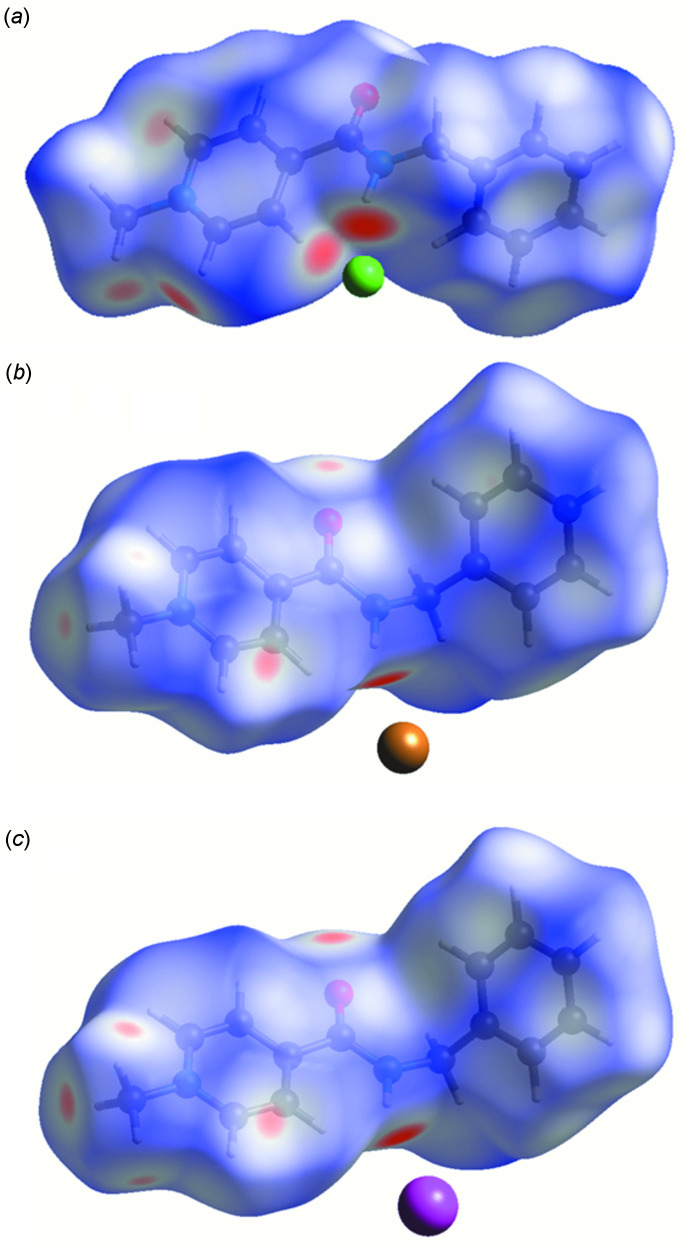
Hirshfeld surfaces of the cation in the (*a*) **AmCl**, (*b*) **AmBr** and (*c*) **AmI** salts mapped over *d*_norm_.

The formation of inter­molecular inter­actions in the two salts under study and the **AmI** salt can be compared using Hirshfeld surface analysis and two-dimensional fingerprint plots [Turner *et al.*, 2017 ▸]. The Hirshfeld surfaces were obtained for the cations using a standard high surface resolution, mapped over *d*_norm_. The red spots on the *d*_norm_ surfaces correspond to contacts that are shorter than the van der Waals radii sum of the closest atoms (Fig. 4 ▸). Such red spots are observed on all the hydrogen atoms participating in the above-mentioned inter­molecular hydrogen bonds (Tables 1 ▸ and 2 ▸). It should be noted that the brightness of the spot on the hydrogen atom decreases with an increase in the radius of the halogen atom, indicating a weakening of the hydrogen bond.

**Figure 5:**
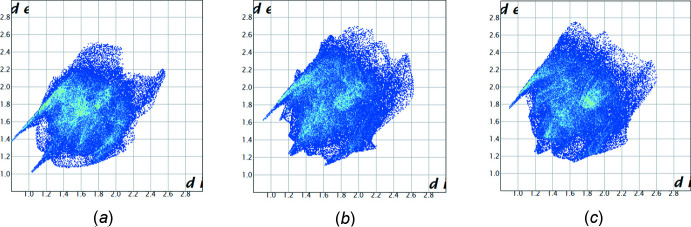
Two-dimensional fingerprint plots for the cation in the three salts under study: (*a*) **AmCl**, (*b*) **AmBr** and (*c*) **AmI**.

**Figure 6:**
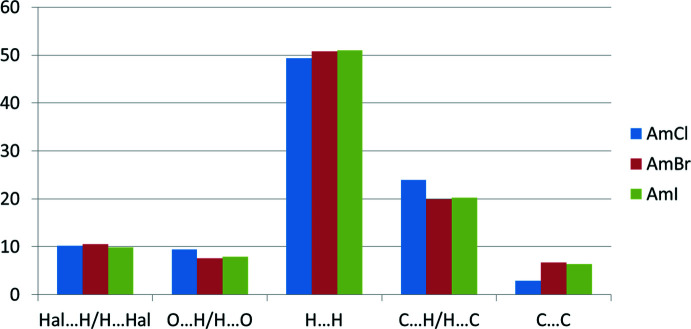
Contributions of the different types of inter­actions to the total Hirshfeld surface of the cation in three halogenide salts.

The hydrogen bonds and short contacts of the cations found in the structures of **AmCl**, **AmBr** and **AmI** are shown in the two-dimensional fingerprint plots presented in Fig. 5 ▸ *a*–*c*. It should be noted that the fingerprint plots constructed for the cations in structures **AmBr** and **AmI** are very similar (Fig. 5 ▸ *b* and 5*c*). The main contribution to the total Hirshfeld surface (49.4% in **AmCl**, 50.8% in **AmBr**, 51.0% in **AmI**) is provided by H⋯H short contacts (Fig. 6 ▸). The contribution of C⋯H/H⋯C contacts is much smaller but also significant (23.9% in **AmCl**, 19.9% in **AmBr**, 20.2% in **AmI**). The similar contributions of Hal⋯H/H⋯Hal contacts (10.2% in **AmCl**, 10.5% in **AmBr**, 9.9% in **AmI**) and O⋯H/H⋯O contacts (9.4% in **AmCl**, 7.6% in **AmBr**, 7.9% in **AmI**) are slightly surprising because of the absence of *X*—H⋯O inter­molecular inter­actions in the structures under study. The presence of two aromatic rings in the cation could result in the formation of stacking inter­actions in the crystal, but the contribution of the C⋯C contacts is the smallest (2.9% in **AmCl**, 6.7% in **AmBr**, 6.4% in **AmI**). The small contribution of the C⋯C contacts agrees with the results of the traditional analysis of inter­molecular inter­actions in a crystal using the shortest distances between atoms belonging to neighbouring mol­ecules (see *Supra­molecular features* section). It should be noted that the contribution of the C⋯C contacts is more than twice as high in the crystals of **AmBr** and **AmI** compared to **AmCl**. This can be explained by a mutual orientation of the pyridine and benzene rings belonging to neighbouring mol­ecules in the **AmBr** and **AmI** crystals. However, there are no effective π–π inter­action between these rings because the distances and angles between the planar π systems are too large.

## Database survey

A search of the Cambridge Structural Database (Version 5.42, update of November 2020; Groom *et al.*, 2016 ▸) revealed the structure of the AmI salt (refcode BEBFIA; Drebushchak *et al.*, 2017 ▸). A comparison with the **AmBr** and **AmI** crystal structures showed that they are isostructural.

## Synthesis and crystallization

The synthesis of salts of 4-[(benzyl­amino)­carbon­yl]-1-methyl­pyridinium halide was carried out according to the reaction scheme below.

**Synthesis and crystallization of AmCl.**

520 mL of aceto­nitrile was cooled to 273–277 K in a glass flask. Chloro­methane (87.8 g, 1.739 mol) was dissolved at this temperature. Benzyl­amide isonicotinic acid (245.78 g, 1.16 mol) and 600 mL of cooled aceto­nitrile and aceto­nitrile solution saturated with chloro­methane were loaded into an autoclave. The autoclave was closed and heated to 373 K. The mixture was incubated for 3 h at this temperature. After that, the mixture was allowed to cool to room temperature. The reaction mixture was transferred into a glass flask and cooled to 273–275 K. The reaction mixture was filtered and the precipitate was rinsed on the filter with 200 mL of cooled aceto­nitrile. The product was dried at 313 K for 12 h. Yield 226 g of crude 4-[(benzyl­amino)­carbon­yl]-1-methyl­pyridinium chloride (75%); white crystals. 
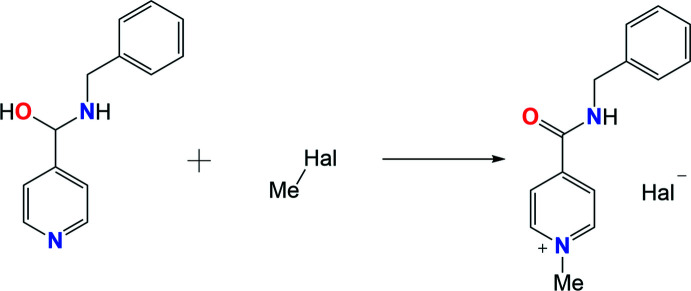


226 g of crude 4-[(benzyl­amino)­carbon­yl]-1-methyl­pyrid­in­ium chloride were dissolved in 265 mL of 90% ethanol and 660 mL of 2-propanol, and 4.25 g activated charcoal were added. The reaction mixture was heated to boiling point, stirred at boiling for 30 min and filtered. The obtained solution was let to spontaneously cool to a temperature of 303 K, then to a temperature of 278–283 K in a cooling water bath, and stirred for 2 h at this temperature. The reaction mixture was filtrated and the precipitate rinsed on the filter with 110 mL of cold 2-propanol. The product was dried at 313 K for 12 h. Yield 180.8 g of 4-[(benzyl­amino)­carbon­yl]-1-methyl­pyrid­in­ium chloride (80%); white crystals; m.p. 474–477 K.

**Synthesis and crystallization of **AmBr**.**

4-[(Benzyl­amino)­carbon­yl]-1-methyl­pyridinium iodide (57.7 g, 0.163 mol), silver bromide (33.77 g, 0.180 mol) and 700 mL of water were loaded into a glass flask. The mixture was stirred for 72 h. The sediment was filtered off. The solvent was evaporated under reduced pressure. 300 mL of aceto­nitrile were added to the precipitate and the mixture was refluxed for 2 h. The reaction mixture was allowed to spontaneously cool to a temperature of 303 K. The reaction mixture was filtered and the precipitate was rinsed on the filter with 50 mL of cold aceto­nitrile. The product was dried at 313 K for 12 h. Yield 14 g of 4-[(benzyl­amino)­carbon­yl]-1-methyl­pyridinium bromide (28%); white crystals; m.p. 465–468 K.

The crystals of **AmCl** and **AmBr** were grown as very small colourless and yellow parallelepipeds, respectively, in contrast to the well-grown yellow block-shaped crystals of **AmI**.

## Spectroscopic characterization

Both salts under consideration were fully characterized by IR, ^1^H NMR and ^13^C NMR spectroscopy. IR spectra of solid samples were acquired on a Thermo Fisher Scientific Nicolet iS50 FTIR spectrometer. ^1^H NMR spectra of samples were measured in DMSO-*d*_6_ on a 600 MHz Varian spectrometer. ^13^C NMR spectra of samples were taken in DMSO-*d*_6_ on a 150 MHz Varian spectrometer.

**Figure 7:**
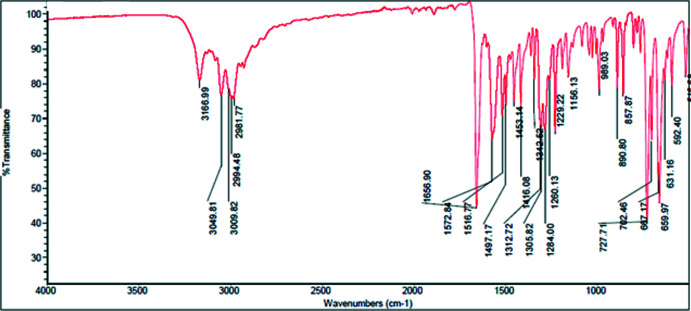
IR spectrum of the **AmCl** salt.

The characteristic vibration frequencies of the main functional groups according to the data of FTIR spectroscopy are shown in Table 3 ▸. The full spectroscopic data are presented below and in Figs. 7 ▸ and 8 ▸. As can be seen from Table 3 ▸, the main difference in IR spectra concerns the valence vibrations of the N—H group and vibrations of C—H bonds in the pyridine ring.

**AmCl**:

IR spectrum (cm^−1^) (Fig. 7 ▸): 592.40, 631.16, 659.97, 667.17, 702.46, 727.71, 857.87, 890.80, 989.03, 1156.13, 1229.22, 1260.13, 1284.00, 1305.82, 1312.72, 1342.62, 1416.08, 1453.14, 1497.17, 1516.77, 1572.84, 1656.90, 2981.77, 2994.48, 3009.82, 3049.81, 3166.99.

^1^H NMR (600 MHz, DMSO-*d*_6_, p.p.m.): δ = 4.40 (*s*, 3H, CH_3_), 4.48–4.49 (*d*, 2H, CH2), 7.21–7.36 (*m*, 5H, Ar), 8.59 (*d*, 2H, Py), 9.21 (*d*, 2H, Py), 10.47 (*s*, H, NH).

^13^C NMR (150 MHz, DMSO-*d*_6_, p.p.m.): δ = 43.42 (CH_2_), 48.37 (CH3), 125.98, 146.94, 147.91 (Py), 127.44, 128.00, 128.75, 139.03 (Ar), 162.12 (C=O).

**Figure 8:**
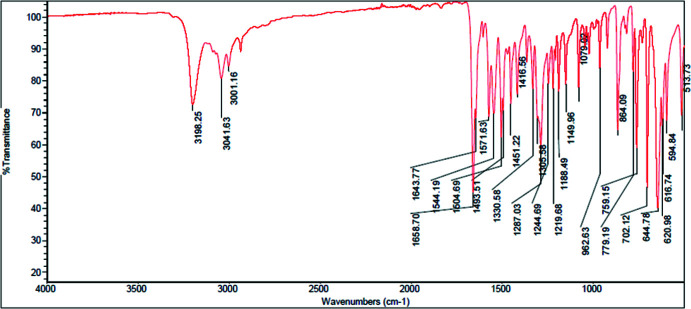
IR spectrum of the **AmBr** salt.

**AmBr**:

IR spectrum (cm^−1^) (Fig. 8 ▸): 594.84, 616.74, 620.98, 644.78, 702.12, 759.15, 779.19, 864.09, 962.63, 1079.02, 1149.96, 1188.49, 1219.68, 1244.69, 1287.03, 1305.58, 1330.58, 1416.56, 1451.22, 1493.51, 1504.69, 1544.19, 1571.63, 1643.77, 1658.70, 3001.16, 3041.63, 3198.25.

^1^H NMR (400 MHz, DMSO-*d*_6_, p.p.m.): δ = 4.41 (*s*, 3H, CH3), 4.51 (*d*, 2H, CH2), 7.23–7.36 (*m*, 5H, Ar), 8.48 (*d*, 2H, Py), 9.21 (*d*, 2H, Py), 9.92 (*s*, H, NH).

^13^C NMR (100 MHz, DMSO-*d*_6_, p.p.m.): δ = 43.52 (CH2), 48.50 (CH_3_), 125.87, 146.99, 147.97 (Py), 127.56, 128.00, 128.84, 138.83 (Ar), 162.31 (C=O).

## Refinement

Crystal data, data collection and structure refinement details are summarized in Table 4 ▸. All of the hydrogen atoms were located in difference-Fourier maps. They were included in calculated positions and treated as riding with C—H = 0.96 Å, *U*_iso_(H) = 1.5*U*_eq_ for methyl groups and with C_ar_—H = 0.93 Å, C*sp* ^2^—H = 0.97 Å, *U*_iso_(H) = 1.2*U*_eq_ for all other hydrogen atoms.

## Supplementary Material

Crystal structure: contains datablock(s) . DOI: 10.1107/S2056989021013505/jq2010sup1.cif

Structure factors: contains datablock(s) AmCl. DOI: 10.1107/S2056989021013505/jq2010AmClsup2.hkl

Structure factors: contains datablock(s) AmBr. DOI: 10.1107/S2056989021013505/jq2010AmBrsup3.hkl

Supporting information file. DOI: 10.1107/S2056989021013505/jq2010AmClsup4.cml

Supporting information file. DOI: 10.1107/S2056989021013505/jq2010AmBrsup5.cml

CCDC references: 2130345, 2130344

Additional supporting information:  crystallographic information; 3D view; checkCIF report

## Figures and Tables

**Table 1:** Hydrogen-bond geometry (Å, °) for **AmCl** [Chem scheme1]

*D*—H⋯*A*	*D*—H	H⋯*A*	*D*⋯*A*	*D*—H⋯*A*
N2—H2⋯Cl1	0.92 (3)	2.26 (3)	3.163 (3)	165 (2)
C1—H1*C*⋯Cl1^i^	0.96	2.89	3.513 (3)	124
C1—H1*A*⋯Cl1^ii^	0.96	2.72	3.633 (3)	160
C2—H2*A*⋯Cl1^ii^	0.93	2.59	3.474 (3)	160
C3—H3⋯Cl1	0.93	2.63	3.531 (3)	165

Symmetry codes: (i) -x+2, -y+1, -z+1; (ii) -x+2, -y+2, -z+1.

**Table 2:** Hydrogen-bond geometry (Å, °) for **AmBr** [Chem scheme1]

*D*—H⋯*A*	*D*—H	H⋯*A*	*D*⋯*A*	*D*—H⋯*A*
N2—H2⋯Br1	0.86	2.68	3.468 (9)	154
C1—H1*A*⋯Br1^i^	0.96	3.04	3.913 (13)	152
C1—H1*C*⋯Br1^ii^	0.96	3.01	3.901 (13)	154

Symmetry codes: (i) x-{\script{1\over 2}}, -y+{\script{1\over 2}}, -z; (ii) x-1, y, z.

**Table 3:** Characteristic vibration frequencies according to the FTIR data

Location of bond	Vibrations	**AmCl**, wavenumbers	**AmBr**, wavenumbers
Valence vibrations of monosubstituted amides	N—H (stretching)	3166	3198
Valence vibrations (aromatic system)	C—H (stretching)	3049	3042
Valence vibrations (CH_3_)	C—H (stretching)	2994	3001
Valence vibrations of the carboxyl group in amides	C=O (stretching)	1656, 1572	1659, 1571
Vibrations of bonds in the pyridine ring	C—H (out-of-plane bending)	727, 659	702, 621

**Table 4:** Experimental details

	**AmCl**	**AmBr**
Crystal data		
Chemical formula	C_14_H_15_N_2_O^+^·Cl^−^	C_14_H_15_N_2_O^+^·Br^−^
*M*_r_	262.73	307.19
Crystal system, space group	Monoclinic, *P*2_1_/*n*	Orthorhombic, *P*2_1_2_1_2_1_
Temperature (K)	293	293
*a*, *b*, *c* (Å)	8.5222 (7), 5.6875 (3), 27.1720 (14)	9.417 (3), 11.099 (5), 14.363 (6)
α, β, γ (°)	90, 91.243 (6), 90	90, 90, 90
*V* (Å^3^)	1316.71 (15)	1501.2 (10)
*Z*	4	4
Radiation type	Mo *K*α	Mo *K*α
μ (mm^−1^)	0.28	2.73
Crystal size (mm)	0.30 × 0.20 × 0.10	0.30 × 0.30 × 0.06

Data collection		
Diffractometer	Xcalibur, Sapphire3	Xcalibur, Sapphire3
Absorption correction	Multi-scan (*CrysAlis PRO*, Rigaku OD 2018 ▸)	Multi-scan (*CrysAlis PRO*, Rigaku OD 2018 ▸)
*T*_min_, *T*_max_	0.624, 1.000	0.068, 1.000
No. of measured, independent and observed [*I* > 2σ(*I*)] reflections	5343, 2302, 1529	10683, 2635, 1583
*R*_int_	0.048	0.118
(sin θ/λ)_max_ (Å^−1^)	0.595	0.594

Refinement		
*R*[*F* ^2^ > 2σ(*F* ^2^)], *wR*(*F* ^2^), *S*	0.051, 0.119, 1.05	0.063, 0.150, 1.00
No. of reflections	2302	2635
No. of parameters	168	164
H-atom treatment	H atoms treated by a mixture of independent and constrained refinement	H-atom parameters constrained
Δρ_max_, Δρ_min_ (e Å^−3^)	0.18, −0.16	0.34, −0.60
Absolute structure	–	Flack *x* determined using 407 quotients [(*I* ^+^)-(*I* ^-^)]/[(*I* ^+^)+(*I* ^-^)] (Parsons et al., 2013 ▸)
Absolute structure parameter	–	0.00 (2)

Computer programs: *CrysAlis PRO* (Rigaku OD, 2018 ▸), *SHELXT2014/5* (Sheldrick, 2015*a* ▸), *SHELXL2016/6* (Sheldrick, 2015*b* ▸), *Mercury* (Macrae *et al.*, 2020 ▸) and *OLEX2* (Dolomanov *et al.*, 2009 ▸).

## supplementary crystallographic information

### 4-[(Benzylamino)carbonyl]-1-methylpyridinium chloride (AmCl) Crystal data

**Table:**

C_14_H_15_N_2_O^+^·Cl^−^	*F*(000) = 552
*M**_r_* = 262.73	*D*_x_ = 1.325 Mg m^−^^3^
Monoclinic, *P*2_1_/*n*	Mo *K*α radiation, λ = 0.71073 Å
*a* = 8.5222 (7) Å	Cell parameters from 766 reflections
*b* = 5.6875 (3) Å	θ = 3.2–20.4°
*c* = 27.1720 (14) Å	µ = 0.28 mm^−^^1^
β = 91.243 (6)°	*T* = 293 K
*V* = 1316.71 (15) Å^3^	Block, colorless
*Z* = 4	0.30 × 0.20 × 0.10 mm

### 4-[(Benzylamino)carbonyl]-1-methylpyridinium chloride (AmCl) Data collection

**Table:**

Xcalibur, Sapphire3 diffractometer	2302 independent reflections
Radiation source: Enhance (Mo) X-ray Source	1529 reflections with *I* > 2σ(*I*)
Detector resolution: 16.1827 pixels mm^-1^	*R*_int_ = 0.048
ω scans	θ_max_ = 25.0°, θ_min_ = 3.0°
Absorption correction: multi-scan (CrysAlisPro, Rigaku OD 2018)	*h* = −10→9
*T*_min_ = 0.624, *T*_max_ = 1.000	*k* = −6→6
5343 measured reflections	*l* = −31→26

### 4-[(Benzylamino)carbonyl]-1-methylpyridinium chloride (AmCl) Refinement

**Table:**

Refinement on *F*^2^	0 restraints
Least-squares matrix: full	Hydrogen site location: mixed
*R*[*F*^2^ > 2σ(*F*^2^)] = 0.051	H atoms treated by a mixture of independent and constrained refinement
*wR*(*F*^2^) = 0.119	*w* = 1/[σ^2^(*F*_o_^2^) + (0.034*P*)^2^] where *P* = (*F*_o_^2^ + 2*F*_c_^2^)/3
*S* = 1.05	(Δ/σ)_max_ < 0.001
2302 reflections	Δρ_max_ = 0.18 e Å^−^^3^
168 parameters	Δρ_min_ = −0.16 e Å^−^^3^

### 4-[(Benzylamino)carbonyl]-1-methylpyridinium chloride (AmCl) Special details

**Table:**

Geometry. All esds (except the esd in the dihedral angle between two l.s. planes) are estimated using the full covariance matrix. The cell esds are taken into account individually in the estimation of esds in distances, angles and torsion angles; correlations between esds in cell parameters are only used when they are defined by crystal symmetry. An approximate (isotropic) treatment of cell esds is used for estimating esds involving l.s. planes.

### 4-[(Benzylamino)carbonyl]-1-methylpyridinium chloride (AmCl) Fractional atomic coordinates and isotropic or equivalent isotropic displacement parameters (Å^2^)

**Table:**

	*x*	*y*	*z*	*U*_iso_*/*U*_eq_	
Cl1	1.00363 (10)	0.87872 (14)	0.60412 (3)	0.0644 (3)	
O1	0.5912 (3)	0.1775 (4)	0.57992 (7)	0.0685 (7)	
N1	0.7393 (3)	0.5404 (4)	0.42221 (8)	0.0507 (6)	
N2	0.7762 (3)	0.4394 (5)	0.60568 (9)	0.0568 (7)	
H2	0.844 (4)	0.561 (5)	0.5990 (10)	0.063 (10)*	
C1	0.7545 (4)	0.6081 (6)	0.37003 (9)	0.0661 (10)	
H1A	0.822085	0.742522	0.367808	0.099*	
H1B	0.652886	0.646109	0.356333	0.099*	
H1C	0.798499	0.479503	0.352058	0.099*	
C2	0.8023 (4)	0.6758 (5)	0.45742 (10)	0.0586 (9)	
H2A	0.855209	0.812634	0.449031	0.070*	
C3	0.7896 (4)	0.6142 (5)	0.50613 (10)	0.0594 (9)	
H3	0.833718	0.709994	0.530460	0.071*	
C4	0.7124 (3)	0.4127 (5)	0.51914 (10)	0.0469 (7)	
C5	0.6493 (4)	0.2765 (5)	0.48176 (10)	0.0551 (8)	
H5	0.595346	0.139381	0.489249	0.066*	
C6	0.6655 (4)	0.3418 (5)	0.43357 (11)	0.0575 (9)	
H6	0.624699	0.246639	0.408589	0.069*	
C7	0.6881 (4)	0.3322 (5)	0.57169 (11)	0.0535 (8)	
C8	0.7728 (4)	0.3762 (5)	0.65733 (9)	0.0603 (9)	
H8A	0.875072	0.408118	0.672190	0.072*	
H8B	0.754012	0.208480	0.659881	0.072*	
C9	0.6503 (4)	0.5038 (5)	0.68622 (9)	0.0492 (8)	
C10	0.5909 (4)	0.7194 (5)	0.67180 (11)	0.0589 (9)	
H10	0.626455	0.789378	0.643179	0.071*	
C11	0.4807 (5)	0.8315 (6)	0.69903 (12)	0.0763 (11)	
H11	0.441586	0.976239	0.688610	0.092*	
C12	0.4267 (5)	0.7325 (7)	0.74176 (13)	0.0789 (11)	
H12	0.349989	0.808053	0.759759	0.095*	
C13	0.4875 (4)	0.5203 (7)	0.75753 (11)	0.0732 (11)	
H13	0.454056	0.453721	0.786750	0.088*	
C14	0.5986 (4)	0.4067 (6)	0.72969 (10)	0.0587 (9)	
H14	0.638986	0.263078	0.740336	0.070*	

### 4-[(Benzylamino)carbonyl]-1-methylpyridinium chloride (AmCl) Atomic displacement parameters (Å^2^)

**Table:**

	*U*^11^	*U*^22^	*U*^33^	*U*^12^	*U*^13^	*U*^23^
Cl1	0.0722 (6)	0.0577 (5)	0.0636 (5)	−0.0109 (5)	0.0095 (4)	−0.0002 (4)
O1	0.0690 (16)	0.0706 (15)	0.0660 (14)	−0.0208 (14)	0.0031 (12)	0.0193 (11)
N1	0.0510 (16)	0.0512 (15)	0.0500 (14)	0.0014 (14)	0.0027 (12)	0.0026 (12)
N2	0.0646 (19)	0.0573 (17)	0.0487 (15)	−0.0113 (16)	0.0033 (14)	0.0087 (13)
C1	0.078 (3)	0.075 (2)	0.0447 (16)	−0.012 (2)	−0.0035 (16)	0.0126 (15)
C2	0.070 (2)	0.0507 (19)	0.0552 (19)	−0.0110 (18)	−0.0021 (17)	0.0057 (15)
C3	0.075 (2)	0.0558 (19)	0.0472 (17)	−0.0142 (19)	−0.0018 (16)	0.0038 (14)
C4	0.0444 (18)	0.0435 (17)	0.0531 (16)	0.0006 (15)	0.0054 (14)	0.0046 (14)
C5	0.055 (2)	0.0516 (18)	0.0588 (18)	−0.0087 (17)	0.0112 (16)	0.0044 (15)
C6	0.062 (2)	0.0515 (19)	0.0585 (19)	−0.0118 (18)	0.0027 (16)	−0.0034 (15)
C7	0.055 (2)	0.0516 (19)	0.0544 (18)	0.0023 (18)	0.0086 (17)	0.0095 (15)
C8	0.064 (2)	0.068 (2)	0.0480 (17)	−0.0007 (19)	−0.0025 (16)	0.0127 (15)
C9	0.056 (2)	0.0489 (18)	0.0422 (15)	−0.0129 (16)	−0.0083 (14)	0.0022 (13)
C10	0.078 (3)	0.0464 (18)	0.0523 (17)	−0.0067 (19)	−0.0081 (17)	0.0038 (15)
C11	0.098 (3)	0.057 (2)	0.073 (2)	0.012 (2)	−0.020 (2)	−0.0109 (18)
C12	0.078 (3)	0.095 (3)	0.064 (2)	0.012 (2)	−0.003 (2)	−0.027 (2)
C13	0.077 (3)	0.094 (3)	0.0485 (18)	−0.012 (2)	0.0034 (19)	−0.0030 (19)
C14	0.069 (2)	0.062 (2)	0.0453 (16)	−0.0060 (19)	−0.0047 (16)	0.0079 (15)

### 4-[(Benzylamino)carbonyl]-1-methylpyridinium chloride (AmCl) Geometric parameters (Å, º)

**Table:**

O1—C7	1.230 (3)	C5—H5	0.9300
N1—C6	1.332 (3)	C6—H6	0.9300
N1—C2	1.332 (4)	C8—C9	1.506 (4)
N1—C1	1.478 (3)	C8—H8A	0.9700
N2—C7	1.326 (4)	C8—H8B	0.9700
N2—C8	1.450 (3)	C9—C10	1.380 (4)
N2—H2	0.92 (3)	C9—C14	1.385 (4)
C1—H1A	0.9600	C10—C11	1.366 (4)
C1—H1B	0.9600	C10—H10	0.9300
C1—H1C	0.9600	C11—C12	1.379 (5)
C2—C3	1.376 (4)	C11—H11	0.9300
C2—H2A	0.9300	C12—C13	1.378 (5)
C3—C4	1.372 (4)	C12—H12	0.9300
C3—H3	0.9300	C13—C14	1.385 (4)
C4—C5	1.377 (4)	C13—H13	0.9300
C4—C7	1.518 (4)	C14—H14	0.9300
C5—C6	1.371 (4)		
			
C6—N1—C2	120.6 (2)	O1—C7—N2	125.0 (3)
C6—N1—C1	119.7 (3)	O1—C7—C4	119.5 (3)
C2—N1—C1	119.7 (3)	N2—C7—C4	115.5 (3)
C7—N2—C8	122.5 (3)	N2—C8—C9	114.5 (2)
C7—N2—H2	123.7 (17)	N2—C8—H8A	108.6
C8—N2—H2	113.8 (17)	C9—C8—H8A	108.6
N1—C1—H1A	109.5	N2—C8—H8B	108.6
N1—C1—H1B	109.5	C9—C8—H8B	108.6
H1A—C1—H1B	109.5	H8A—C8—H8B	107.6
N1—C1—H1C	109.5	C10—C9—C14	118.4 (3)
H1A—C1—H1C	109.5	C10—C9—C8	122.3 (3)
H1B—C1—H1C	109.5	C14—C9—C8	119.3 (3)
N1—C2—C3	120.3 (3)	C11—C10—C9	120.9 (3)
N1—C2—H2A	119.9	C11—C10—H10	119.6
C3—C2—H2A	119.9	C9—C10—H10	119.6
C4—C3—C2	120.6 (3)	C10—C11—C12	120.8 (3)
C4—C3—H3	119.7	C10—C11—H11	119.6
C2—C3—H3	119.7	C12—C11—H11	119.6
C3—C4—C5	117.5 (3)	C13—C12—C11	119.3 (3)
C3—C4—C7	124.8 (3)	C13—C12—H12	120.3
C5—C4—C7	117.7 (3)	C11—C12—H12	120.3
C6—C5—C4	120.4 (3)	C12—C13—C14	119.7 (3)
C6—C5—H5	119.8	C12—C13—H13	120.1
C4—C5—H5	119.8	C14—C13—H13	120.1
N1—C6—C5	120.6 (3)	C13—C14—C9	120.9 (3)
N1—C6—H6	119.7	C13—C14—H14	119.5
C5—C6—H6	119.7	C9—C14—H14	119.5
			
C6—N1—C2—C3	−1.2 (5)	C3—C4—C7—N2	−14.7 (4)
C1—N1—C2—C3	−179.7 (3)	C5—C4—C7—N2	166.9 (3)
N1—C2—C3—C4	0.2 (5)	C7—N2—C8—C9	−88.1 (4)
C2—C3—C4—C5	0.1 (5)	N2—C8—C9—C10	−24.3 (4)
C2—C3—C4—C7	−178.3 (3)	N2—C8—C9—C14	157.9 (3)
C3—C4—C5—C6	0.6 (5)	C14—C9—C10—C11	−1.7 (5)
C7—C4—C5—C6	179.1 (3)	C8—C9—C10—C11	−179.5 (3)
C2—N1—C6—C5	1.9 (5)	C9—C10—C11—C12	0.4 (5)
C1—N1—C6—C5	−179.6 (3)	C10—C11—C12—C13	1.4 (6)
C4—C5—C6—N1	−1.6 (5)	C11—C12—C13—C14	−1.7 (6)
C8—N2—C7—O1	3.0 (5)	C12—C13—C14—C9	0.3 (5)
C8—N2—C7—C4	−177.1 (2)	C10—C9—C14—C13	1.4 (5)
C3—C4—C7—O1	165.2 (3)	C8—C9—C14—C13	179.2 (3)
C5—C4—C7—O1	−13.3 (4)		

### 4-[(Benzylamino)carbonyl]-1-methylpyridinium chloride (AmCl) Hydrogen-bond geometry (Å, º)

**Table:**

*D*—H···*A*	*D*—H	H···*A*	*D*···*A*	*D*—H···*A*
N2—H2···Cl1	0.92 (3)	2.26 (3)	3.163 (3)	165 (2)
C1—H1*C*···Cl1^i^	0.96	2.89	3.513 (3)	124
C1—H1*A*···Cl1^ii^	0.96	2.72	3.633 (3)	160
C2—H2*A*···Cl1^ii^	0.93	2.59	3.474 (3)	160
C3—H3···Cl1	0.93	2.63	3.531 (3)	165

Symmetry codes: (i) −*x*+2, −*y*+1, −*z*+1; (ii) −*x*+2, −*y*+2, −*z*+1.

### 4-[(Benzylamino)carbonyl]-1-methylpyridinium bromide (AmBr) Crystal data

**Table:**

C_14_H_15_N_2_O^+^·Br^−^	*D*_x_ = 1.359 Mg m^−^^3^
*M**_r_* = 307.19	Mo *K*α radiation, λ = 0.71073 Å
Orthorhombic, *P*2_1_2_1_2_1_	Cell parameters from 748 reflections
*a* = 9.417 (3) Å	θ = 3.2–24.8°
*b* = 11.099 (5) Å	µ = 2.73 mm^−^^1^
*c* = 14.363 (6) Å	*T* = 293 K
*V* = 1501.2 (10) Å^3^	Plate, yellow
*Z* = 4	0.30 × 0.30 × 0.06 mm
*F*(000) = 624	

### 4-[(Benzylamino)carbonyl]-1-methylpyridinium bromide (AmBr) Data collection

**Table:**

Xcalibur, Sapphire3 diffractometer	2635 independent reflections
Radiation source: Enhance (Mo) X-ray Source	1583 reflections with *I* > 2σ(*I*)
Detector resolution: 16.1827 pixels mm^-1^	*R*_int_ = 0.118
ω scans	θ_max_ = 25.0°, θ_min_ = 3.4°
Absorption correction: multi-scan (CrysAlisPro, Rigaku OD 2018)	*h* = −11→11
*T*_min_ = 0.068, *T*_max_ = 1.000	*k* = −13→13
10683 measured reflections	*l* = −17→16

### 4-[(Benzylamino)carbonyl]-1-methylpyridinium bromide (AmBr) Refinement

**Table:**

Refinement on *F*^2^	Hydrogen site location: inferred from neighbouring sites
Least-squares matrix: full	H-atom parameters constrained
*R*[*F*^2^ > 2σ(*F*^2^)] = 0.063	*w* = 1/[σ^2^(*F*_o_^2^) + (0.0446*P*)^2^] where *P* = (*F*_o_^2^ + 2*F*_c_^2^)/3
*wR*(*F*^2^) = 0.150	(Δ/σ)_max_ < 0.001
*S* = 1.00	Δρ_max_ = 0.34 e Å^−^^3^
2635 reflections	Δρ_min_ = −0.60 e Å^−^^3^
164 parameters	Absolute structure: Flack *x* determined using 407 quotients [(*I*^+^)-(*I*^-^)]/[(*I*^+^)+(*I*^-^)] (Parsons et al., 2013)
0 restraints	Absolute structure parameter: 0.00 (2)

### 4-[(Benzylamino)carbonyl]-1-methylpyridinium bromide (AmBr) Special details

**Table:**

Geometry. All esds (except the esd in the dihedral angle between two l.s. planes) are estimated using the full covariance matrix. The cell esds are taken into account individually in the estimation of esds in distances, angles and torsion angles; correlations between esds in cell parameters are only used when they are defined by crystal symmetry. An approximate (isotropic) treatment of cell esds is used for estimating esds involving l.s. planes.

### 4-[(Benzylamino)carbonyl]-1-methylpyridinium bromide (AmBr) Fractional atomic coordinates and isotropic or equivalent isotropic displacement parameters (Å^2^)

**Table:**

	*x*	*y*	*z*	*U*_iso_*/*U*_eq_	
Br1	0.91899 (12)	0.26554 (10)	0.17853 (8)	0.0849 (5)	
O1	0.5589 (10)	0.6049 (7)	0.3657 (5)	0.091 (3)	
N2	0.7250 (9)	0.4630 (7)	0.3158 (7)	0.073 (2)	
H2	0.743714	0.406610	0.276630	0.088*	
N1	0.3008 (10)	0.4355 (9)	0.0832 (7)	0.073 (2)	
C7	0.5970 (12)	0.5238 (9)	0.3083 (7)	0.070 (3)	
C3	0.5277 (11)	0.3994 (10)	0.1584 (8)	0.071 (3)	
H3	0.612742	0.356784	0.159577	0.085*	
C2	0.4254 (14)	0.3763 (9)	0.0884 (9)	0.078 (3)	
H2A	0.445066	0.317583	0.043984	0.093*	
C5	0.3663 (13)	0.5481 (12)	0.2211 (9)	0.090 (4)	
H5	0.343497	0.606658	0.264908	0.108*	
C4	0.4986 (13)	0.4888 (10)	0.2269 (8)	0.071 (3)	
C6	0.2692 (14)	0.5207 (11)	0.1512 (10)	0.095 (4)	
H6	0.181979	0.559919	0.149850	0.114*	
C9	0.8166 (12)	0.4132 (10)	0.4771 (8)	0.073 (3)	
C8	0.8319 (12)	0.4919 (11)	0.3897 (9)	0.090 (4)	
H8A	0.821881	0.575832	0.407276	0.108*	
H8B	0.926489	0.481169	0.364293	0.108*	
C13	0.6899 (13)	0.3640 (12)	0.6235 (8)	0.085 (4)	
H13	0.616600	0.379851	0.665032	0.102*	
C14	0.7079 (11)	0.4343 (11)	0.5440 (8)	0.073 (3)	
H14	0.645855	0.498173	0.534234	0.088*	
C12	0.7868 (15)	0.2666 (12)	0.6398 (9)	0.098 (4)	
H12	0.776537	0.217390	0.691806	0.118*	
C1	0.1934 (12)	0.4075 (14)	0.0071 (9)	0.106 (5)	
H1A	0.227194	0.341433	−0.029942	0.158*	
H1B	0.180938	0.477098	−0.031718	0.158*	
H1C	0.104155	0.386183	0.034937	0.158*	
C10	0.9118 (15)	0.3165 (10)	0.4951 (9)	0.096 (4)	
H10	0.984059	0.299441	0.453014	0.115*	
C11	0.8973 (18)	0.2467 (12)	0.5761 (10)	0.122 (5)	
H11	0.962607	0.185674	0.587752	0.147*	

### 4-[(Benzylamino)carbonyl]-1-methylpyridinium bromide (AmBr) Atomic displacement parameters (Å^2^)

**Table:**

	*U*^11^	*U*^22^	*U*^33^	*U*^12^	*U*^13^	*U*^23^
Br1	0.0782 (7)	0.0895 (8)	0.0868 (8)	0.0122 (7)	0.0041 (7)	0.0018 (7)
O1	0.106 (6)	0.085 (5)	0.083 (6)	0.013 (5)	−0.001 (6)	−0.023 (4)
N2	0.077 (6)	0.074 (6)	0.068 (6)	−0.001 (5)	−0.002 (6)	−0.003 (5)
N1	0.063 (6)	0.077 (7)	0.080 (7)	0.004 (5)	−0.002 (5)	0.000 (5)
C7	0.084 (7)	0.066 (7)	0.060 (7)	0.000 (6)	0.011 (7)	0.014 (5)
C3	0.071 (7)	0.072 (7)	0.071 (8)	0.006 (5)	−0.005 (6)	0.005 (5)
C2	0.077 (7)	0.069 (7)	0.087 (8)	−0.003 (7)	0.020 (8)	−0.004 (5)
C5	0.086 (8)	0.098 (10)	0.086 (9)	0.027 (7)	−0.006 (8)	−0.027 (7)
C4	0.075 (7)	0.071 (8)	0.066 (9)	−0.006 (6)	0.005 (7)	0.004 (6)
C6	0.072 (8)	0.103 (10)	0.111 (12)	0.025 (7)	0.001 (8)	−0.007 (8)
C9	0.078 (8)	0.066 (7)	0.074 (8)	0.000 (6)	−0.012 (7)	0.001 (6)
C8	0.074 (7)	0.102 (10)	0.094 (10)	−0.019 (7)	0.005 (7)	0.000 (7)
C13	0.084 (8)	0.104 (10)	0.065 (8)	−0.015 (8)	0.007 (7)	−0.008 (7)
C14	0.064 (7)	0.082 (8)	0.075 (8)	0.004 (6)	−0.010 (7)	−0.009 (7)
C12	0.126 (10)	0.095 (10)	0.073 (8)	−0.011 (9)	−0.024 (8)	0.007 (8)
C1	0.082 (8)	0.140 (12)	0.095 (10)	−0.004 (9)	−0.009 (8)	−0.014 (9)
C10	0.095 (9)	0.104 (9)	0.088 (9)	0.032 (9)	−0.004 (9)	0.006 (7)
C11	0.178 (15)	0.099 (10)	0.091 (9)	0.059 (12)	−0.027 (11)	−0.014 (8)

### 4-[(Benzylamino)carbonyl]-1-methylpyridinium bromide (AmBr) Geometric parameters (Å, º)

**Table:**

O1—C7	1.273 (12)	C9—C14	1.423 (14)
N2—C7	1.386 (12)	C9—C8	1.536 (15)
N2—C8	1.498 (14)	C8—H8A	0.9700
N2—H2	0.8600	C8—H8B	0.9700
N1—C2	1.347 (14)	C13—C14	1.392 (15)
N1—C6	1.392 (14)	C13—C12	1.433 (16)
N1—C1	1.521 (13)	C13—H13	0.9300
C7—C4	1.540 (15)	C14—H14	0.9300
C3—C2	1.416 (15)	C12—C11	1.404 (18)
C3—C4	1.425 (14)	C12—H12	0.9300
C3—H3	0.9300	C1—H1A	0.9600
C2—H2A	0.9300	C1—H1B	0.9600
C5—C6	1.392 (16)	C1—H1C	0.9600
C5—C4	1.412 (14)	C10—C11	1.404 (17)
C5—H5	0.9300	C10—H10	0.9300
C6—H6	0.9300	C11—H11	0.9300
C9—C10	1.422 (15)		
			
C7—N2—C8	122.4 (9)	N2—C8—C9	113.2 (9)
C7—N2—H2	118.8	N2—C8—H8A	108.9
C8—N2—H2	118.8	C9—C8—H8A	108.9
C2—N1—C6	118.6 (11)	N2—C8—H8B	108.9
C2—N1—C1	121.3 (10)	C9—C8—H8B	108.9
C6—N1—C1	120.0 (10)	H8A—C8—H8B	107.7
O1—C7—N2	122.6 (11)	C14—C13—C12	118.6 (11)
O1—C7—C4	120.0 (10)	C14—C13—H13	120.7
N2—C7—C4	117.4 (10)	C12—C13—H13	120.7
C2—C3—C4	119.1 (10)	C13—C14—C9	123.3 (11)
C2—C3—H3	120.5	C13—C14—H14	118.4
C4—C3—H3	120.5	C9—C14—H14	118.4
N1—C2—C3	122.9 (10)	C11—C12—C13	118.9 (12)
N1—C2—H2A	118.5	C11—C12—H12	120.6
C3—C2—H2A	118.5	C13—C12—H12	120.6
C6—C5—C4	121.4 (11)	N1—C1—H1A	109.5
C6—C5—H5	119.3	N1—C1—H1B	109.5
C4—C5—H5	119.3	H1A—C1—H1B	109.5
C5—C4—C3	117.0 (11)	N1—C1—H1C	109.5
C5—C4—C7	117.3 (10)	H1A—C1—H1C	109.5
C3—C4—C7	125.7 (11)	H1B—C1—H1C	109.5
C5—C6—N1	121.0 (11)	C11—C10—C9	120.4 (12)
C5—C6—H6	119.5	C11—C10—H10	119.8
N1—C6—H6	119.5	C9—C10—H10	119.8
C10—C9—C14	117.0 (11)	C10—C11—C12	121.7 (13)
C10—C9—C8	121.2 (11)	C10—C11—H11	119.2
C14—C9—C8	121.8 (10)	C12—C11—H11	119.2
			
C8—N2—C7—O1	−2.8 (15)	C2—N1—C6—C5	2.6 (18)
C8—N2—C7—C4	177.7 (9)	C1—N1—C6—C5	−179.7 (13)
C6—N1—C2—C3	−2.0 (17)	C7—N2—C8—C9	93.5 (12)
C1—N1—C2—C3	−179.7 (10)	C10—C9—C8—N2	103.8 (12)
C4—C3—C2—N1	0.2 (16)	C14—C9—C8—N2	−76.9 (13)
C6—C5—C4—C3	−0.3 (19)	C12—C13—C14—C9	1.1 (17)
C6—C5—C4—C7	−178.7 (11)	C10—C9—C14—C13	−1.4 (16)
C2—C3—C4—C5	0.9 (16)	C8—C9—C14—C13	179.3 (10)
C2—C3—C4—C7	179.1 (10)	C14—C13—C12—C11	0.9 (17)
O1—C7—C4—C5	−1.4 (16)	C14—C9—C10—C11	−0.3 (18)
N2—C7—C4—C5	178.0 (10)	C8—C9—C10—C11	179.1 (12)
O1—C7—C4—C3	−179.6 (10)	C9—C10—C11—C12	2 (2)
N2—C7—C4—C3	−0.2 (15)	C13—C12—C11—C10	−3 (2)
C4—C5—C6—N1	−1 (2)		

### 4-[(Benzylamino)carbonyl]-1-methylpyridinium bromide (AmBr) Hydrogen-bond geometry (Å, º)

**Table:**

*D*—H···*A*	*D*—H	H···*A*	*D*···*A*	*D*—H···*A*
N2—H2···Br1	0.86	2.68	3.468 (9)	154
C1—H1*A*···Br1^i^	0.96	3.04	3.913 (13)	152
C1—H1*C*···Br1^ii^	0.96	3.01	3.901 (13)	154

Symmetry codes: (i) *x*−1/2, −*y*+1/2, −*z*; (ii) *x*−1, *y*, *z*.
